# Error Detection in Emergency Radiology Reports Using a Large Language Model: Multistage Evaluation Study

**DOI:** 10.2196/86841

**Published:** 2026-04-14

**Authors:** Hui Shen, Tianyang Wu, Fei Wang, Jin Fang, Yuange Li, Xiaoling Wu, Shuyi Liu, Liting Chen, Qiuping Ren, Xiao Meng, Jiatong Xu, Jie Sun, Yujie Zhao, Xin Liu, Liaoyuan Wang, Guipeng Mai, Jingjing You, Zhe Jin, Xuewei Wu, Wenle He, Xue Han, Shuixing Zhang, Dong Zeng, Bin Zhang

**Affiliations:** 1 Department of Radiology, The First Affiliated Hospital of Jinan University, No. 613 Huangpu West Road, Tianhe, Guangzhou, Guangdong, 510630, China, 86 15217921427; 2 School of Biomedical Engineering, Southern Medical University, Guangzhou, Guangdong, China; 3 Department of Radiology, The Affiliated Hospital of Guangdong Medical University, Zhanjiang, Guangdong, China; 4 Department of Radiology, Guangzhou Women and Children's Medical Center, Guangzhou Medical University, Guangzhou, Guangdong, China; 5 Department of Radiology, Nanfang Hospital, Southern Medical University, Guangzhou, Guangdong, China; 6 Department of Radiology, Longhu District People's Hospital of Shantou, Shantou, Guangdong, China

**Keywords:** emergency radiology, large language models, error detection, quality control

## Abstract

**Background:**

Emergency radiology requires highly accurate reporting under time constraints; yet, increasing workloads raise the risk of errors. While large language models (LLMs) show potential for proofreading in general radiology, their performance in emergency settings and non-English contexts remains unclear.

**Objective:**

We aim to evaluate the performance of a domain-optimized LLM, DeepSeek-R1, for identifying errors in Chinese emergency radiology reports, with comparison against assessments by board-certified radiologists.

**Methods:**

We compiled 7435 emergency reports (dataset 1; radiography, computed tomography, and magnetic resonance imaging) collected from November 2024 to April 2025. In stage 1, a total of 5 LLMs were evaluated using 200 reports. The best model, DeepSeek-R1, proceeded to stages 2 and 3, where 0-shot and few-shot learning were tested on a separate set (n=100). Model performance was compared against 12 radiologists. Stage 4 validated real-world utility on 800 verified reports.

**Results:**

In subdataset 1, under stress-testing conditions, DeepSeek-R1 achieved a higher error detection rate in the few-shot setting than in the 0-shot setting (84.4% vs 60.9%, *P*=.003). Its performance exceeded that of radiology residents (84.4% vs 51.6% and 53.1%, respectively; both *P*<.05) and showed no statistically significant difference compared with senior radiologists and attending radiologists (84.4% vs 68.8%‐93.8%, *P*=.26 to ≥.99). Compared with residents, DeepSeek-R1 detected more critical omissions (100% vs 25% and 50%; both *P*<.05) and other errors (92% vs 33% and 33%; both *P*=.02). In dataset 2, collected from independent institutions, DeepSeek-R1 achieved a detection rate of 95% under the few-shot setting. Reading time was shorter than that of human readers (92 vs 109 s). In real-world validation, DeepSeek-R1 identified 117 true reporting errors, yielding a positive predictive value of 56.5%.

**Conclusions:**

DeepSeek-R1 holds promise for improving quality control in emergency radiology reports. Its performance and efficiency support its use as an assistive proofreading tool in real-world radiology workflows.

## Introduction

Emergency radiology reports are critical for guiding timely and accurate patient management, particularly in trauma, acute illness, and other time-sensitive clinical scenarios. Errors in these reports, ranging from factual inaccuracies (eg, laterality confusion) to interpretive inconsistencies, can directly impact patient outcomes, leading to misdiagnoses, delayed treatments, or inappropriate management [1-3]. The growing volume of emergency cases, coupled with radiologist shortages and high workloads, further increases the likelihood of mistakes [4-7]. Traditional proofreading methods, such as double-reading by senior radiologists, are effective but time-consuming and often impractical in fast-paced emergency settings [8].

Recent advances in large language models (LLMs) provide promising solutions for automating error detection [9,10]. Studies have shown that LLMs such as GPT-4 (OpenAI Inc) and Claude 3.5 Sonnet (Anthropic PBC) can identify inconsistencies in radiology reports with accuracy comparable to human experts [11-13]. For instance, GPT-4 achieved an error detection rate of 82.7% in general radiology reports, comparable to senior radiologists (89.3%) while reducing reading time from 25.1 to 3.5 seconds per report [12]. Similarly, Claude 3.5 Sonnet outperformed radiologists in detecting factual errors in head computed tomography (CT) reports, with a sensitivity of 89% compared to 33%‐69% among human readers [13]. In Chinese ultrasound reports, LLMs show promise in identifying spelling and logical errors, with Claude 3.5 Sonnet achieving a 52.3% detection rate in 0-shot settings [14]. Despite these advances, several critical gaps remain. First, LLM performance in emergency radiology, a domain characterized by fragmented information, urgent decision-making, and complex multimodal findings, remains unexplored. Second, most studies used synthetically generated errors or single-institution data**,** limiting generalizability to real-world emergency scenarios [12,13]. Third, while GPT-4 and Claude show strengths in English contexts, their efficacy in non-English emergency reports (eg, Chinese) is suboptimal due to linguistic nuances and terminology variations [15,16]. DeepSeek-R1, a state-of-the-art LLM specifically optimized for Chinese clinical text, offers a valuable opportunity to address these limitations [17,18]. Its architecture integrates domain-specific pretraining on multilingual clinical corpora, potentially enhancing error detection in non-English emergency reports [19]. However, rigorous validation using real-world emergency radiology data is still lacking.

Therefore, this study aims to evaluate DeepSeek-R1’s ability to identify errors in real-world Chinese emergency radiology reports, benchmark its performance against both other LLMs and radiologists of varying experience levels, and assess its potential for integration into clinical workflows. With a focus on practical applicability, this work strives to establish a new benchmark for artificial intelligence (AI)–assisted quality assurance in emergency radiology.

## Methods

### Ethical Considerations

This retrospective study was approved by the Ethics Review Board of the First Affiliated Hospital of Jinan University (20250306). The requirement for informed consent was waived due to this study’s retrospective nature. All patient data used during this study were strictly anonymized. No personally identifiable information was disclosed to any LLMs, thereby ensuring patient privacy and adherence to ethical standards. Participants received no compensation.

### Dataset and Error Categories

A total of 7435 medical records between November 2024 and April 2025 were collected from the First Affiliated Hospital of Jinan University. The dataset 1 consisted of 3 types of emergency radiology reports: CT (n=5237), magnetic resonance imaging (MRI; n=381), and radiography (n=1817). Each report included 4 sections: patient information, examination items, findings, and impression. To increase the diversity of the dataset, dataset 2 consisted of 50 error-free emergency radiology reports that passed quality control and 50 erroneous reports that did not meet quality control, collected from 2 hospitals: Nanfang Hospital, Southern Medical University, and the Affiliated Hospital of Guangdong Medical University. Both unreviewed and senior physician-reviewed versions are available for each record. All patient identifiers were anonymized before inclusion in the dataset.

First, a total of 200 error-free reports were randomly selected from dataset 1 to form a subdataset 1 using a freely available research data randomization tool [20], all of which were verified by human experts to ensure accuracy. These reports served as the basis for evaluating the error detection capabilities of both LLMs and physicians. Then, artificial errors were introduced into 100 of these reports through a randomized process. Informed by previous studies on common error types in radiology reports [21-23], the error categories included (1) omission: the omission of relevant words or phrases, including both deletions and missing words (eg, “fracture” instead of “no fracture”); (2) insertion: the unintentional insertion of incorrect words or phrases, including inappropriate terms, substitutions, insertions, or word confusions (eg, “abnormal” instead of “normal”); (4) spelling: Chinese spelling errors that may arise from rapid typing using Pinyin input methods (eg, “纵隔” misspelled as “纵膈,” “椎体” misspelled as “锥体”); (5) side confusion: laterality errors (eg, “right” instead of “left,” “lateral” instead of “medial”); and (6) other errors: errors not fitting the above categories, such as incorrect dates, errors in image or series numbering, unit discrepancies (eg, centimeter vs millimeter), template-related inaccuracies, and punctuation errors. Error definitions, applications, and distribution of each error type are detailed in Table 1, and Figures S1 and S2 in Multimedia Appendix 1.

**Table 1.** Types of errors, their descriptions, and examples used in this study. The examples presented in the table are actual errors identified by DeepSeek-R1 from original emergency radiology reports of the First Affiliated Hospital of Jinan University, with the erroneous portions shown in italics.

Error type	Description	Example	
		Finding	Impression
Omission	The omission of relevant words or phrases, including both deletions and missing words	There is no evidence of hemorrhage, edema, mass effect, or infarction. “*The visualized bilateral maxillary sinuses show mucosal thickening with patchy fluid density shadows within.*”	*Normal study*
Insertion	The unintentional insertion of incorrect words or phrases, including inappropriate terms, substitutions, insertions, or word confusions	There is a fracture at the inferior pole of the patella with displacement of the larger fragment inferiorly and anteriorly, and displacement of a smaller bony fragment inferiorly. Alignment within the right knee joint is appropriate. No further pathologic step-offs or interruptions of the cortex are delineated. The bone trabeculae are homogeneously structured.	*Nondisplaced* fracture at the inferior pole of the patella.
Spelling	Chinese spelling errors that may arise from rapid typing using Pinyin input methods	Disruption of cortical continuity is noted in the distal phalanx of the left *hallux* (*拇趾*)*,* with an identifiable transverse or radiolucent fracture line.	Mildly displaced fracture of the distal phalanx of the left *hallux* (*拇指*), *which is a spelling error in Chinese.*
Side confusion	Laterality errors	Adjacent to the middle phalanx of the *left* fifth finger, there is a small, well-defined osseous density fragment.	Small osseous density adjacent to the *right* fifth middle phalanx, suspicious for an avulsion fracture.
Other errors	Errors not fitting the above categories, such as incorrect dates, errors in image or series numbering, unit discrepancies, template-related inaccuracies, and punctuation errors	The liver demonstrates smooth contours and normal proportions. Multiple rounds of low-attenuation lesions are present within the hepatic parenchyma, with well-defined margins. The largest lesion measures approximately *1.2 cm* in diameter.	Multiple low-attenuation hepatic lesions, the largest measuring approximately *1.2 mm*.

Clinical experts methodically implanted 127 errors into the modified reports, with no more than 2 errors per report. Therefore, a comparative dataset of 100 error-free and 100 erroneous reports was established to evaluate the diagnostic discrepancy detection capabilities of both LLMs and radiologists. Only the errors intentionally inserted into the text were used as the reference standard. To ensure the absence of any additional errors in the radiology reports, 3 readers (HS, XW, and BZ, with 5, 5, and 8 years of experience, respectively) independently reviewed the reports. Discrepancies were resolved via consensus review among 3 readers.

Two types of prompts were developed for the LLMs: 0-shot prompts and few-shot prompts. The few-shot prompt was constructed by incorporating 6 additional emergency radiology report samples into the 0-shot prompt framework, while maintaining consistency across all other prompt components. These 6 example reports were randomly selected from the pool of emergency radiology reports, with deliberate attention to ensuring diagnostic diversity and coverage of common error types, including item omission, insertion, spelling errors, laterality confusion, and other frequently encountered reporting mistakes. This stratified yet random selection strategy was intended to reflect the variability of real-world radiology reporting without biasing the model toward any specific diagnosis or error category. The anonymized example reports used for few-shot prompting are provided in Table S1 in Multimedia Appendix 1. Each prompt was structured into three main sections: (1) role context and task description, (2) definitions of report error types, and (3) output constraints (Table 2). In the 0-shot setting, the prompt was specifically designed with an emphasis on Chinese medical terminology to ensure alignment with the linguistic and domain-specific characteristics of emergency radiology reports in clinical practice. While this domain-specific tailoring was intended to improve task relevance, we acknowledge that it may introduce a performance advantage for DeepSeek-R1, which has been trained on corpora with similar linguistic and clinical characteristics.

**Table 2.** Detailed prompts and parameters of large language models used in this study.

Prompt name	Prompt text
0-shot prompt	You are a radiology physician. In the subsequent text, I will provide you with an emergency radiology report containing the following three sections: “Examination details” “Findings” and “Impression”, please assess the report for potential inaccuracies. If you identify any of the following error types, please address the corresponding issues. Omission: Discrepancies between imaging findings and imaging diagnosis that may lead to ambiguity, missed diagnosis, or misdiagnosis. For example, a positive lesion mentioned in imaging findings is not referred in the imaging diagnosis. Insertion: Unintentional inclusion of incorrect terms or phrases within imaging findings or radiological diagnosis, such as improper word substitutions or insertions. Spelling errors: Spelling mistakes caused by rapid typing of chinese characters, such as substitutions of homophones that may alter the intended meaning. Positional discrepancies: Inconsistencies between imaging descriptions and diagnosis regarding anatomical locations, such as confusion between right and left or medial and lateral positions. Other errors: Errors not falling into the above categories, including inaccuracies in report comparison dates, measurement units, punctuation, etc Please review each report provided below one by one to check for errors. Output your findings in the following format: “Serial number XXX /n Error categories: −1-, −2-, … /n Cause of error: …”
Few-shot prompt	I am going to provide you with six example reports: one error-free report and five reports containing the various categories of errors listed. The sole purpose of these example reports is to improve your comprehension and to help you recognize the various error categories mentioned in the subsequent tasks. We then entered each report and its corresponding error description and categorization in order. For example, one example report contained “Side confusion,” specifically, the radiology description showed a small, well-defined osseous density fragment in the left fifth finger, whereas the radiology impression described small osseous density adjacent to the right fifth middle phalanx, suspicious for an avulsion fracture. At the end of the learning phase containing all 6 example reports, we entered “provided example reports completed” to indicate the end of the examples. We then followed the prompts mentioned in the 0-shot setting to begin error detection on the test set reports.

### Study Design

**Figure 1.**
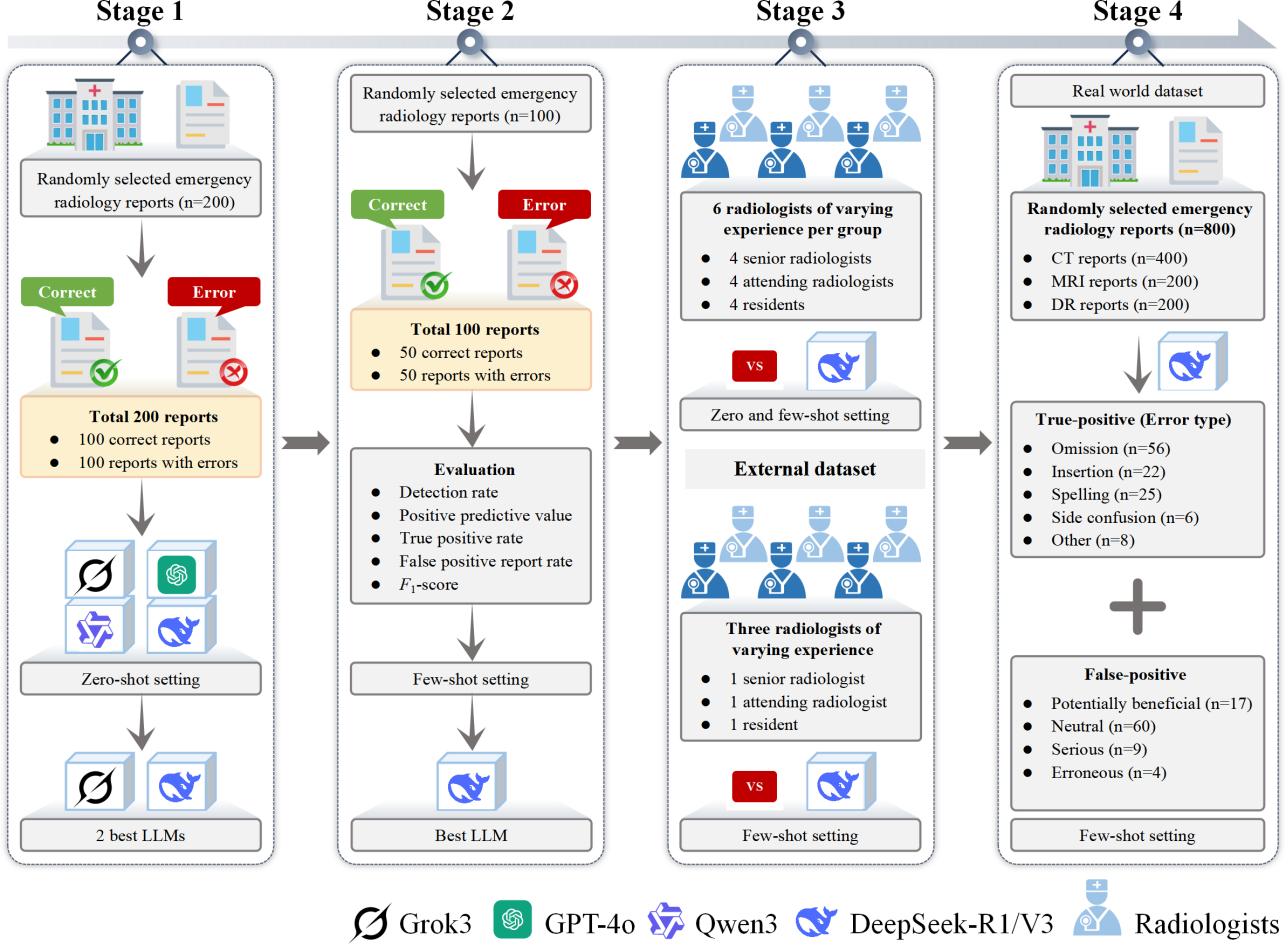
Workflow of the multistage validation study. Schematic diagram of the 4-stage experimental design. (**A**) Initial screening of 5 large language models using 200 reports (100 error-free; 100 with 127 synthetic errors across 5 categories). (**B**) Few-shot optimization of top-performing models (DeepSeek-R1) using clinical examples. (**C**) Benchmarking against varying experience of radiologists (seniors, attendings, and residents) in the 0 and few-shot settings on subdataset 1 and subdataset 2. (**D**) Real-world validation on 800 verified emergency reports. CT: computed tomography; DR: digital radiography; LLM: large language model; MRI: magnetic resonance imaging.

This study evaluated the capabilities of different LLMs and radiologists in detecting errors in emergency radiology reports across diverse scenarios through a 4-stage experimental design (Figure 1).

### Stage 1: Initial Model Screening

First, 200 error-free reports were randomly selected from dataset 1 using a freeware data randomization tool [20], and then proofread by human experts to ensure accuracy. We evaluated 5 LLMs (open-source: DeepSeek-R1, DeepSeek-V3, and Qwen3-235B; closed-source: GPT-4o and Grok3) using 0-shot prompts to rank their performance in detecting errors in emergency radiology reports (200 cases), with the aim of determining the 2 best-performing models for subsequent analysis.

### Stage 2: Few-Shot Prompt Evaluation

The 2 top-performing LLMs from stage 1 were re-evaluated in 0-shot and few-shot settings using a test set (100 reports and 64 errors), which was composed by randomly selecting 50 correct reports and 50 artificial-error subgroup reports. This phase explored whether the error detection capabilities of the optimal model improve in a few-shot setting and determined the best-performing model for stage 3 and stage 4 analysis.

### Stage 3: Exploratory Analysis of DeepSeek-R1 Performance

Using the top-performance model (DeepSeek-R1) from stage 2 and using the same test set as stage 2 (100 reports and 64 errors), the error detection performance and time taken by 12 radiologists of different experience levels (4 senior radiologists, 4 attending radiologists, and 4 residents) were assessed via a customized online survey platform [24]. Experts were stratified into 2 groups (6 experts per group) and each group independently evaluated the 100 reports, benchmarking against the LLM’s performance under simulated 0-shot and few-shot workflows. In addition, under the few-shot workflow, further testing was conducted on dataset 2 (100 reports containing 60 errors) to compare the error detection performance between DeepSeek-R1 and 3 radiologists with different experience levels (1 senior radiologist, 1 attending radiologist, and 1 resident). Stages 2 and 3 were intentionally designed as stress-testing phases for boundary performance, in which error prevalence was artificially enriched to enable controlled and statistically efficient comparison of error detection sensitivity across models and human readers.

### Stage 4: Validation on a Real-World Dataset

Using the few-shot model from stage 2, DeepSeek-R1 processed 800 unverified real emergency radiology reports (400 CT, 200 MR, and 200 radiograph reports), with outputs compared to final reviewed versions. Reports were then assessed by 2 senior radiologists to determine if LLM outputs could influence final diagnoses and sorted as either true errors or false-positive results. These assessments quantified how AI assistance might impact false-positive generation. The clinical impact of false-positive responses was further assessed to determine their actual harmfulness or potential benefits, with the detailed criteria outlined in Table S2 in Multimedia Appendix 1.

### Statistical Analysis

All emergency report evaluation results were collected and analyzed using R (version 4.2.3; R Foundation) software. We measured the number of correctly identified errors, mean processing time, and associated costs. The average detection time and associated costs per report were calculated from evaluations of 20 randomly selected emergency radiology reports. Only artificially introduced errors during data processing were included in the measurements. To ensure the reliability of our findings, all reports in the dataset and their corresponding outputs were reviewed by 3 reviewers (HS, TW, and BZ). Detailed methods for error quantification and analytical procedures are provided in the text and Table S3 in Multimedia Appendix 1.

We compared the number of correctly identified errors and mean processing time between LLMs and radiologists using metrics such as positive predictive value (PPV), true positive rate (TPR), false positive report rate (FPRR), and *F*_1_-score. We computed CIs using the Wilson score method [25]. To compare performance metrics between radiologists and LLMs across 0-shot and few-shot settings, Wald chi-square tests were used to evaluate differences in error detection metrics (PPV, TPR, and *F*_1_-scores) and FPRR. Bonferroni correction adjusted for multiple comparisons, with a 2-tailed *P* value <.05 considered statistically significant. Cohen κ assessed agreement between model predictions and individual readers, while the intraclass correlation coefficient measured interrater reliability among radiologists. Interreader reliability was assessed using Cohen κ, with the following classification: 0.01‐0.20 (negligible to slight), 0.21‐0.40 (fair), 0.41‐0.60 (moderate), 0.61‐0.80 (substantial), and 0.81‐1.00 (nearly perfect) [26].

## Results

### Stage 1: Performance in Detecting Errors Among LLMs

In stage 1 (200 reports and 127 errors), DeepSeek-R1 achieved the optimal performance, with a detection rate of 51.2%. For error detection, the PPV, TPR, and *F*_1_-score were 64.4% (95% CI 55%‐74%), 51.2% (95% CI 42.3%‐60%), and 57% (95% CI 49%‐60%), respectively. Grok3 was the second-best performing model, with a detection rate of 48% (61/127). For error detection, the PPV, TPR, and *F*_1_-score were 64.9% (95% CI 54.9%‐74.7%), 48% (95% CI 39.5%‐56.9%), and 55.2% (95% CI 47.1‐62.8), respectively. In contrast, DeepSeek-V3 yielded a detection rate of 37% (47/127), with PPV and TPR of 54% (95% CI 43.4%‐64.4%) and 37% (95% CI 28.8%‐45.7%), respectively. Additionally, Qwen3 and GPT-4o achieved a detection rate of only 33.1% (42/127), with TPR of 33.1% (95% CI 24.8%‐41.2%), while with PPV of 48.3% (95% CI 37.3%‐58.8%) and 50% (95% CI 39.2%‐60.8%), *F*_1_-score of 39.3% (95% CI 30.7%‐47.2%) and 39.8% (95% CI 31.1%‐47.8%), respectively (Table 3).

**Table 3.** Performance of different LLMs^a^ in error detection in a 0-shot setting. Bonferroni correction was used to correct *P* values for multiple comparisons with DeepSeek-R1. Higher values of detection rate, PPV^b^, and TPR^c^ indicate better detection performance of the model, while a higher FPRR^d^ value suggests poorer detection performance.

Model	Detection rate (%)		*P* value	PPV (%), 95% CI	*P* value	TPR (%), 95% CI	*P* value	*F*_1_-score (%), 95% CI	*P* value	FPRR (%), 95% CI	*P* value
	Detection rate (%), 95% CI	Values, n/N									
DeepSeek-R1	51.2 (40.9-62.2)	65/127	—^e^	64.4 (55.0-74.0)	—	51.2 (42.3-60.0)	—	57.0 (49.0-64.5)	—^f^	16.5 (11.5-22.0)	—
Grok3	48.0 (37.8-58.3)	61/127	≥.99	64.9 (54.9-74.7)	≥.99	48.0 (39.5-56.9)	≥.99	55.2 (47.1-62.8)	≥.99	16.5 (11.5-22.0)	≥.99
DeepSeek-V3	37.0 (27.6-47.2)	47/127	.09	54.0 (43.4-64.4)	.35	37.0 (28.8-45.7)	.09	43.9 (35.3-52.2)	.19	20.0 (14.5-26.0)	≥.99
Qwen3	33.1 (24.4-42.5)	42/127	.01^f^	48.3 (37.3-58.8)	.052	33.1 (25.2-41.4)	.01^f^	39.3 (30.7-47.2)	.03^f^	22.5 (17.0-28.5)	.52
GPT-4o	33.1 (24.4-42.5)	42/127	.01^f^	50.0 (39.2-60.8)	.10	33.1 (24.8-41.2)	.01^f^	39.8 (31.1-47.8)	.03^f^	21.0 (15.5-27.0)	≥.99

a LLMs: large language models.

b PPV: positive predictive value.

c TPR: true positive rate.

d FPRR: false positive report rate.

e Not available.

f *P* < 0.05 was considered statistically significant.

Regarding the negative impact, the false positives generated by DeepSeek-R1 and Grok3 were comparable. For example, the FPRR for DeepSeek-R1 and grok3 both was 16.5% (95% CI 11.5%‐22%). In contrast, DeepSeek-V3, Qwen3, and GPT-4o generated significantly more false positives, with an FPRR of 20% (95% CI 14.5%‐26%), 22.5% (95% CI 17%‐28.5%), and 21% (95% CI 15.5%‐27%), respectively, with no statistically significant differences (*P*=.52 to ≥.99; Table 3).

### Stages 2 and 3: Exploratory Analysis of DeepSeek-R1 Performance: Overall Performance in Detecting Errors on Subdataset 1 (100 Reports With 64 Errors)

In stage 2, we compared the performance of DeepSeek-R1 and Grok3 under both 0-shot and few-shot settings against radiologists with varying experience levels on dataset 1 (100 reports with 64 errors). Both models showed a higher error detection rate in the few-shot setting compared to the 0-shot setting. It should be noted that these detection rates reflect sensitivity under controlled, error-enriched conditions and are not intended to represent expected detection performance in routine clinical practice. Specifically, DeepSeek-R1’s detection rate increased significantly from 60.9% (39/64) to 84.4% (54/64; *P*=.003), whereas Grok3’s improvement was not statistically significant, rising from 53.1% (34/64) to 56.3% (36/64; *P*=.72). A significant performance difference was observed between DeepSeek-R1 and Grok3 in the few-shot setting (*P*<.001), but not in the 0-shot setting (*P*=.38).

In the 0-shot error detection task, DeepSeek-R1’s detection rate (60.9%) was significantly higher than that of resident 2 (60.9% vs 35.9%, *P*<.05). However, there was no evidence of a difference in the percentage of detected errors between DeepSeek-R1 and the other radiologists per report (*P* value range, .15 to ≥.99). Under the few-shot learning setting, DeepSeek-R1 achieved a detection rate of 84.4% (54/64), surpassing the few-shot performance of resident radiologists. However, there was no evidence of a difference in the percentage of detected errors between DeepSeek-R1 and the other radiologists per report (*P* value range, .26 to ≥.99). Detailed performance metrics for LLMs and radiologists are provided in Table 4.

**Table 4.** Comparison of error detection between LLMs^a^ and the radiologists in 0-shot and few-shot settings. Bonferroni correction was used to correct *P* values for multiple comparisons.^b^

Model	Detection rate (%)		*P* value	PPV^c^ (%), 95% CI	*P* value	TPR^d^ (%), 95% CI	*P* value	*F*_1_-score (%), 95% CI	*P* value	FPRR^e^ (%), 95% CI	*P* value
	Detection rate (%), 95% CI	Values, n/N									
0-shot											
DeepSeek-R1	60.9 (47.9-72.6)	39/64	Ref.^f^	90.7 (81.3-97.9)	Ref.	60.9 (49.2-72.6)	Ref.	72.9 (63.0-81.7)	Ref.	4.0 (1.0-8.0)	Ref.
Grok3	53.1 (40.3-65.6)	34/64	≥.99	79.1 (63.5-89.4)	.89	53.1 (40.7-65.0)	≥.99	63.6 (52.0-73.4)	.90	9.0 (4.0-15.0)	≥.99
Senior 1	73.4 (60.7-83.4)	47/64	.92	90.4 (81.5-97.8)	>.99	73.4 (60.7-83.4)	.90	81.0 (72.2-88.3)	≥.99	5.0 (1.0-10.0)	≥.99
Senior 2	71.9 (59.1-82.1)	46/64	≥.99	93.9 (86.3-100.0)	>.99	71.9 (60.3-82.8)	≥.99	81.4 (72.7-88.9)	.87	3.0 (0.0-7.0)	≥.99
Attending 1	68.8 (55.8-79.2)	44/64	≥.99	86.3 (75.9-94.8)	>.99	68.8 (56.9-79.7)	≥.99	76.5 (67.2-84.4)	≥.99	7.0 (3.0-13.0)	≥.99
Attending 2	65.6 (52.6-76.8)	42/64	≥.99	84.0 (73.5-93.6)	>.99	65.6 (53.5-76.8)	≥.99	73.7 (63.5-82.1)	≥.99	8.0 (3.0-13.0)	≥.99
Resident 1	40.6 (28.8-53.6)	26/64	.15	92.9 (81.8-100.0)	>.99	40.6 (29.0-53.1)	.13	56.5 (43.8-68.0)	.06	2.0 (0.0-5.0)	≥.99
Resident 2	35.9 (24.6-49.0)	23/64	.03^g^	85.2 (70.4-96.8)	>.99	35.9 (24.6-48.3)	.021^g^	50.5 (37.2-62.7)	<.001^g^	4.0 (1.0-8.0)	≥.99
Few-shot											
DeepSeek-R1	84.4 (72.7-91.9)	54/64	Ref.	91.5 (83.6-98.2)	Ref.	84.4 (75.0-92.6)	Ref.	87.8 (81.1-93.3)	Ref.	5.0 (1.0-10.0)	Ref.
Grok3	56.3 (43.3-68.4)	36/64	<.001^g^	82.9 (70.3-93.9)	≥.99	56.3 (42.2-66.7)	.001	66.0 (54.2-75.9)	<.001**^g^**	7.0 (2.0-12.0)	≥.99
Senior 3	93.8 (84.0-98.0)	60/64	.63	96.8 (91.5-100.0)	≥.99	93.8 (87.3-98.6)	.60	95.2 (91.1-98.5)	.30	2.0 (0.0-5.0)	≥.99
Senior 4	84.4 (72.7-91.9)	54/64	≥.99	93.1 (85.7-98.5)	≥.99	84.4 (75.4-93.0)	≥.99	88.5 (82.1-94.1)	≥.99	4.0 (1.0-8.0)	≥.99
Attending 3	70.3 (57.4-80.8)	45/64	.40	91.8 (83.3-98.1)	≥.99	70.3 (58.6-81.3)	.38	79.6 (70.7-87.2)	.65	4.0 (1.0-8.0)	≥.99
Attending 4	68.8 (55.8-79.4)	44/64	.26	91.7 (83.0-98.1)	≥.99	68.8 (57.6-79.7)	.27	78.6 (69.8-86.4)	.42	4.0 (1.0-8.0)	≥.99
Resident 3	53.1 (40.3-65.6)	34/64	<.001^g^	89.5 (78.4-97.6)	>.99	53.1 (40.9-65.5)	<.001^g^	66.7 (55.2-76.5)	.001^g^	4.0 (1.0-8.0)	≥.99
Resident 4	51.6 (38.8-64.1)	33/64	<.001^g^	86.8 (75.0-97.1)	>.99	51.6 (39.7-63.8)	<.001^g^	64.7 (53.3-74.8)	<.001^g^	5.0 (1.0-10.0)	≥.99

a LLM: large language model.

b The performance of DeepSeek-R1 in detecting errors was compared with that of Grok3 and radiologists using Wald chi-square tests. Higher values of detection rate, positive predictive value, and true positive rate indicate better detection performance of the model, while a higher false positive report rate value suggests poorer detection performance.

c PPV: positive predictive value.

d TPR: true positive rate.

e FPRR: false positive report rate.

f Ref.: Reference.

g *P* < 0.05 was considered statistically significant.

### Performance in Detecting Errors by Error Type on Subdataset 1 (100 Reports With 64 Errors)

**Figure 2.**
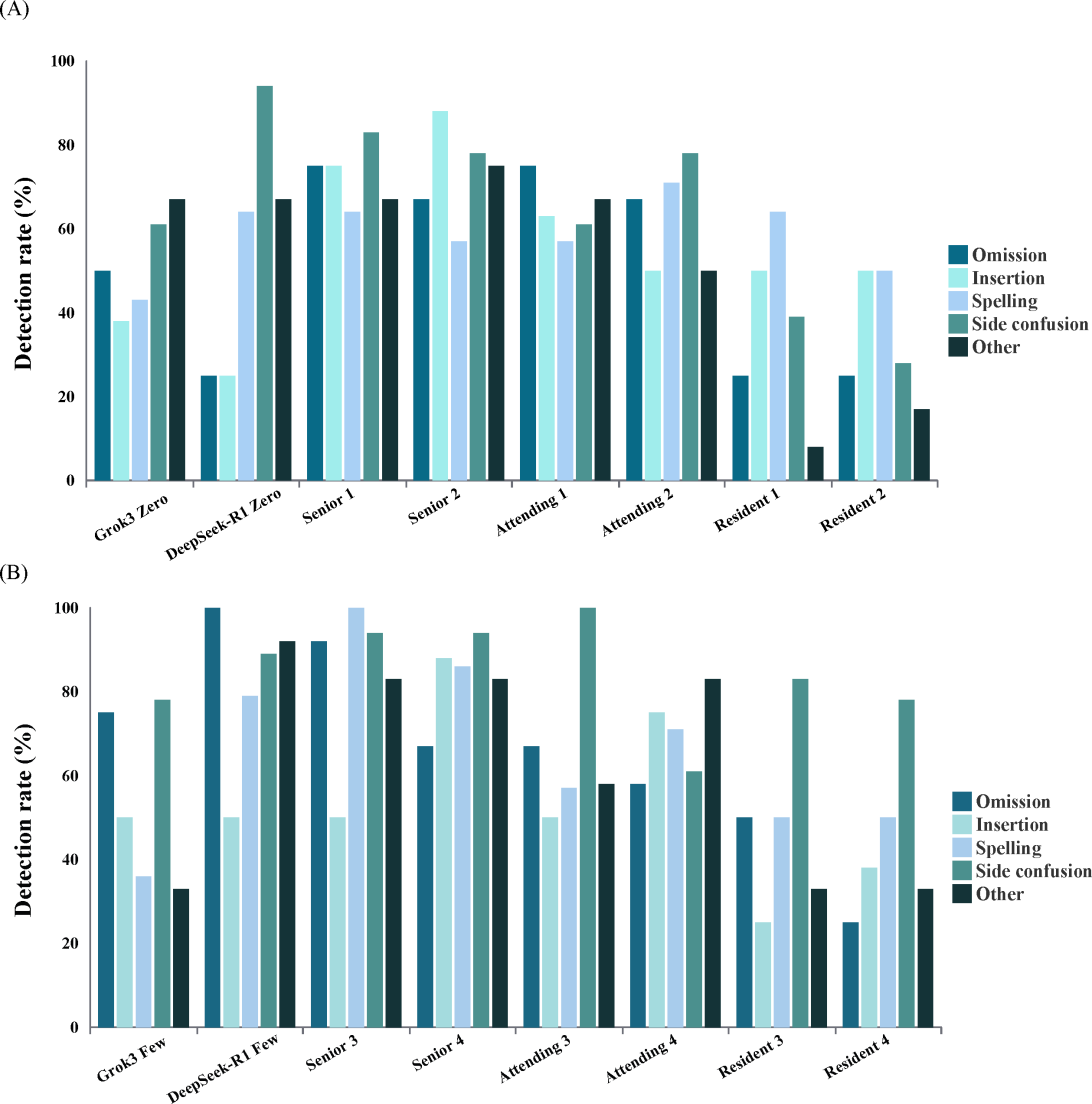
Performance comparison for critical error types. Bar graphs comparing proofreading performance between human readers and large language models across error types. (**A**) Detection rates in a 0-shot setting. (**B**) Detection rates in a few-shot setting.

In the 0-shot setting, DeepSeek-R1 showed superior performance over 2 residents in detecting side confusion, with a detection rate of 94% (95% CI 71‐100) compared to 39% (95% CI 18‐64) and 28% (95% CI 11‐54; *P*=.003 and <.001, respectively; Figure 2A and Table 5). In addition, DeepSeek-R1 detected other types of errors more frequently than resident 1 (67%, 95% CI 35‐89 vs 8%, 95% CI 1‐40; *P*=.022). However, no significant differences were observed between DeepSeek-R1 and the other radiologists in the overall error detection rate per report (*P* values range from .091 to >.99).

**Table 5.** Comparison of detection rates for different error types in radiology reports in 0-shot and few-shot settings. Other errors included errors that did not fit into the defined categories, such as incorrect date entries, errors in numbering of images and/or series, and mistakes in specifying units of measurement (eg, centimeter vs millimeter). Bonferroni correction was used to correct *P* values for multiple comparisons.^a^

	Omission		*P* value	Insertion		*P* value	Spelling		*P* value	Side confusion		*P* value	Other		*P* value
	Detection rate (%), 95% CI	Values, n/N		Detection rate (%), 95% CI	Values, n/N		Detection rate (%), 95% CI	Values, n/N		Detection rate (%), 95% CI	Values, n/N		Detection rate (%), 95% CI	Values, n/N	
0-shot															
DeepSeek-R1	25 (7-57)	3/12	Ref.^b^	25 (45-64)	2/8	Ref.	64 (36-86)	9/14	Ref.	94 (71-100)	17/18	Ref.	67 (35-89)	8/12	Ref.
Grok3	50 (22-77)	6/12	≥.99	38 (10-74)	3/8	≥.99	43 (19-70)	6/14	≥.99	61 (36-82)	11/18	.11	67 (35-89)	8/12	≥.99
Senior 1	75 (43-93)	9/12	.10	75 (36-96)	6/8	.32	64 (36-86)	9/14	≥.99	83 (58-96)	15/18	≥.99	67 (35-89)	8/12	≥.99
Senior 2	67 (35-89)	8/12	.28	88 (47-99)	7/8	.08	57 (30-81)	8/14	≥.99	78 (52-93)	14/18	≥.99	75 (43-93)	9/12	≥.99
Attending 1	75 (43-93)	9/12	.10	63 (26-90)	5/8	.91	57 (30-81)	8/14	≥.99	61 (36-82)	11/18	.11	67 (35-89)	8/12	≥.99
Attending 2	67 (35-89)	8/12	.28	50 (17-83)	4/8	≥.99	71 (42-90)	10/14	≥.99	78 (52-93)	14/18	≥.99	50 (22-78)	6/12	≥.99
Resident 1	42 (19-68)	5/12	≥.99	50 (17, 83)	4/8	≥.99	64 (36-86)	9/14	≥.99	39 (18-64)	7/18	.003^c^	8 (1-40)	1/12	.02^c^
Resident 2	42 (19-68)	5/12	≥.99	50 (17-83)	4/8	≥.99	50 (24-76)	7/14	≥.99	28 (11-54)	5/18	<.001^c^	17 (3-50)	2/12	.09
Few-shot															
DeepSeek-R1	100 (70-100)	12/12	Ref.	50 (22-77)	4/8	Ref.	79 (49-94)	11/14	Ref.	89 (64-98)	16/18	Ref.	92 (60-100)	11/12	Ref.
Grok3	75 (43-93)	9/12	.45	50 (22-77)	4/8	≥.99	36 (14-64)	5/14	.15	78 (52-93)	14/18	≥.99	33 (11-65)	4/12	.02^c^
Senior 3	92 (60-99)	11/12	≥.99	100 (68-100)	8/8	.15	100 (73-100)	14/14	.47	94 (71-100)	17/18	≥.99	83 (51-97)	10/12	≥.99
Senior 4	67 (35-89)	8/12	.20	88 (47-99)	7/8	.74	86 (56-97)	12/14	≥.99	94 (71-100)	17/18	≥.99	83 (51-97)	10/12	≥.99
Attending 3	67 (35-89)	8/12	.20	50 (17-83)	4/8	≥.99	57 (30-81)	8/14	≥.99	100 (78-100)	18/18	≥.99	58 (29-84)	7/12	.42
Attending 4	58 (29-84)	7/12	.08	75 (36-96)	6/8	≥.99	71 (42-90)	10/14	≥.99	61 (36-82)	11/18	.38	83 (51-97)	10/12	≥.99
Resident 3	50 (22-77)	6/12	.03^c^	25 (45-64)	2/8	≥.99	50 (24-76)	7/14	.80	83 (58-96)	15/18	≥.99	33 (11-65)	4/12	.02^c^
Resident 4	42 (19-68)	5/12	.01^c^	38 (10-74)	3/8	≥.99	50 (24-76)	7/14	.80	78 (52-93)	14/18	≥.99	33 (11-65)	4/12	.02^c^

a The number of errors correctly detected by DeepSeek-R1 was compared with that of Grok3 and radiologists by using Wald chi-square tests.

b Ref.: Reference.

c *P*<.05 was considered statistically significant.

In the few-shot setting, DeepSeek-R1 outperformed resident 3 in detecting both omission errors (detection rate, 100%, 95% CI 70‐100 vs 50%, 95% CI 22-77 and 92%, 95% CI 60‐100 vs 33%, 95% CI 11‐65; *P*=.03 and *P*=.02, respectively) and resident 4 (100%, 95% CI 70‐100 vs 25%, 95% CI 7‐57 and 92%, 95% CI 60‐100 vs 33%, 95% CI 11‐65; *P*=.01 and *P*=.02, respectively). However, given the limited sample size, the subgroup analyses were likely underpowered, and the resulting comparisons should be viewed as exploratory and interpreted with caution. More results are displayed in Figure 2B and Table 5.

### Performance in Detecting Errors by Imaging Modality on Subdataset 1 (100 Reports With 64 Errors)

In the 0-shot learning scenario, no significant difference was observed in error detection between DeepSeek-R1 and the radiologists for either radiography reports (detection rate: 66.7% vs 41.9%‐87.1%; *P* value range: .29 to >.99) or CT/MRI reports (45.5% vs 27.3%‐69.7%; *P* value range: .17 to >.99).

In the few-shot learning scenario, DeepSeek-R1 detected significantly more errors than the lowest-performing resident radiologist across both radiography and CT/MRI reports. For radiography reports, the detection rate was 90.3% (95% CI 73.1‐97.4) compared to 54.8% (95% CI 36.3‐72.2; *P*=.01). For CT/MRI reports, DeepSeek-R1 achieved a detection rate of 78.8% (95% CI 60.6‐90.4) versus 45.4% (95% CI 28.5‐63.4; *P*=.04). However, no significant differences were observed between DeepSeek-R1 and the other radiologists in error detection rates per report (*P* values ranging from .053 to >.99 for radiography and .07 to >.99 for CT/MRI). Additional results are provided in Table 6.

**Table 6.** Subgroup analyses of imaging modalities.

	Total		*P* value	Radiography		*P* value	CT^a^ and MRI^b^		*P* value
	Detection rate (%), 95% CI	Values, n/N		Detection rate (%), 95% CI	Values, n/N		Detection rate (%), 95% CI	Values, n/N	
0-shot									
DeepSeek-R1	60.9 (47.9-72.6)	39/64	Ref.^c^	66.7 (45.4-80.2)	21/31	Ref.	54.5 (38.0-70.2)	18/33	Ref.
Grok3	53.1 (40.3-65.6)	34/64	>.99	64.5 (46.9-78.9)	20/31	>.99	42.4 (27.2-59.2)	14/33	>.99
Senior 1	73.4 (60.7-83.4)	47/64	.92	87.1 (69.2-95.8)	27/31	.48	60.6 (36.6-71.5)	20/33	>.99
Senior 2	71.9 (59.1-82.1)	46/64	>.99	74.2 (55.1-87.5)	23/31	>.99	69.7 (51.1-83.8)	23/33	>.99
Attending 1	68.8 (55.8-79.2)	44/64	>.99	77.4 (58.5-89.7)	24/31	>.99	60.6 (42.2-76.6)	20/33	>.99
Attending 2	65.6 (52.6-76.8)	42/64	>.99	74.2 (55.4-87.5)	23/31	>.99	57.6 (39.4-74.1)	19/33	>.99
Resident 1	40.6 (28.8-53.6)	26/64	.15	41.9 (52.1-60.7)	13/31	.29	33.3 (18.6-51.9)	11/33	.58
Resident 2	35.9 (24.6-49.0)	23/64	.03^d^	45.2 (27.8-63.7)	14/31	.51	27.3 (13.9-45.8)	9/33	.17
Few-shot									
DeepSeek-R1	84.4 (72.7-91.9)	54/64	Ref.	90.3 (73.1-97.4)	28/31	Ref.	78.8 (60.6-90.4)	26/33	Ref.
Grok3	56.3 (43.3-68.4)	36/64	.003^d^	66.7 (45.8-82.7)	21/31	.20	45.4 (28.5-63.4)	15/33	.04^d^
Senior 3	93.8 (84.0-98.0)	60/64	.63	93.5 (77.2-98.9)	29/31	>.99	93.9 (78.4-98.9)	31/33	.51
Senior 4	84.4 (72.7-91.9)	54/64	>.99	93.5 (77.2-99.0)	29/31	>.99	75.8 (57.4-88.3)	25/33	>.99
Attending 3	70.3 (57.4-80.8)	45/64	.40	77.4 (58.5-89.7)	24/31	>.99	63.6 (45.2-79.0)	21/33	>.99
Attending 4	68.8 (55.8-79.4)	44/64	.26	67.7 (48.5-82.7)	21/31	.20	69.7 (51.1-83.8)	23/33	>.99
Resident 3	53.1 (40.3-65.6)	34/64	<.001^d^	61.3 (42.3-77.6)	19/31	.053	45.4 (28.5-63.4)	15/33	.04^d^
Resident 4	51.6 (38.8-64.1)	33/64	<.001^d^	54.8 (36.3-72.2)	17/31	.01^d^	48.5 (31.2-66.1)	16/33	.07

a CT: computed tomography.

b MRI: magnetic resonance imaging.

c Ref.: Reference.

d *P*<.05 was considered statistically significant.

### Performance in Detecting Errors on Dataset 2 (100 Reports With 60 Errors)

In the dataset 2 under the few-shot setting, DeepSeek-R1 achieved a detection rate of 95%, which was significantly higher than that of resident 5 (61.7%, *P*<.001) and attending radiologist 5 (71.7%, *P*=.002). These results suggest that DeepSeek-R1’s performance is consistent across different institutions and report structures.

### Stage 4: Validation on a Real-World Dataset

Among the 800 reports analyzed, Deepseek-R1 classified 207 reports as having errors, among which 117 were true errors, yielding a PPV of 0.565. The distribution of error types in the real-world dataset showed that omission was the most common error (n=56), whereas incorrect laterality was the least common (n=6). Furthermore, Deepseek-R1 successfully detected at least one instance of every error category.

The clinical impact of false-positive results incorrectly classified by Deepseek-R1 was further evaluated. There were no reports in which the model introduced errors based on content not present in the original report. Most serious false-positive results involved the addition of clinically insignificant observations to the impression section. Importantly, these outputs did not involve hallucinated or fabricated findings but instead reflected oversensitivity, such as suggestions to include clinically low-impact observations. This pattern of over-alerting, rather than content invention, is likely to preserve radiologist trust in the system. Moreover, approximately 19% of false-positive alerts were considered potentially beneficial for improving report clarity or completeness, thereby mitigating the negative psychological impact typically associated with false alarms.

In the analysis of false-positive results, 17 (18.9%) report changes were identified as potentially beneficial. The improvements were as follows: correcting typographical and grammatical errors (n=1); enhancing oversimplified impressions to improve clarity (n=6); elaborating on observations that were mentioned in the impressions but insufficiently detailed in the findings section, leading to more thorough documentation (n=3); and adding clinically relevant details, such as nerve root compressions, air-fluid levels, spinal stenosis, or new observations, that were documented in the findings but omitted from the impressions, thereby improving the completeness of the report (n=7).

### Interrater Agreement

**Figure 3.**
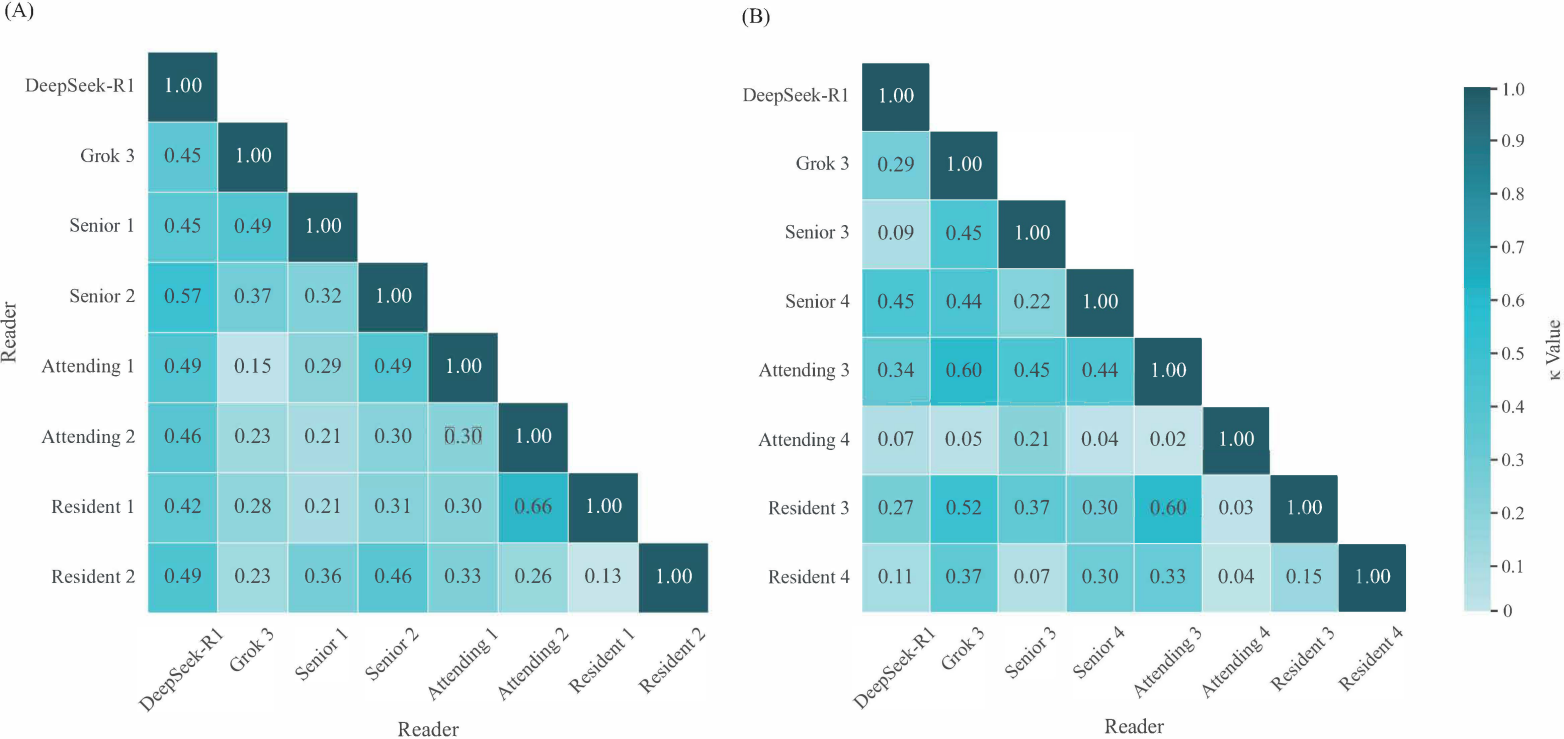
Interrater agreement analysis. Cohen κ coefficients for pairwise agreement among readers (large language models and radiologists). (A) Consistency for error types in the 0-shot setting. (B) Consistency for error types in the few-shot setting. Agreement scale: –1: complete inconsistency; 0: occasional agreement; 0.01-0.20: slight agreement; 0.21-0.40: fair agreement; 0.41-0.60: moderate agreement; 0.61-0.80: substantial agreement; and 0.81-1.00: almost perfect agreement.

Figure 3A,B, Tables S4 and S5 in Multimedia Appendix 1 display the heatmap of Cohen κ coefficients illustrating pairwise agreement among readers in detecting errors in radiology reports under the 0-shot and few-shot settings. The analysis involved 12 radiologists (4 seniors, 4 attendings, and 4 residents) and 2 AI models (DeepSeek-R1 and Grok3). The interrater agreement analysis revealed several key patterns. Between the 2 AI systems in the 0-shot and few-shot settings, DeepSeek-R1 and Grok3 exhibited moderate and fair agreement (κ=0.45 and 0.29, respectively). Among human raters, agreement ranged from slight to substantial, with the lowest consistency observed between the 2 attendings (κ=0.02) under the 0-shot setting and the highest between attending 2 and resident 1 (κ=0.66) under the few-shot setting. In terms of human-AI agreement, DeepSeek-R1 showed fair to moderate consistency with radiologists (κ=0.42‐0.57) under the 0-shot setting, while under the few-shot setting, agreement ranged from slight to moderate (κ=0.09‐0.45). This pattern of variability in human interpretations, contrasted with the more consistent AI-human agreement, highlights both the subjective nature of error detection in radiology reports and the potential of AI systems to provide more standardized assessments. The generally low to moderate agreement among all raters (κ=0.02‐0.66) underscores the complexity and inherent subjectivity in radiology report error detection, suggesting that even experienced clinicians may apply different criteria when evaluating report accuracy.

### Reading Time

In the task of error detection for 100 reports, LLMs demonstrated significant time efficiency advantages. Grok3 processed 100 reports in 0.34 and 0.26 hours under 0-shot and few-shot settings, respectively. For the same task, DeepSeek-R1 required 2.56 hours in the 0-shot setting and 2.26 hours in the few-shot setting.

In contrast, radiologists’ reading times ranged from 3.04 to 5.36 hours. For individual report reading, LLMs exhibited even more pronounced speed: Grok3 required only 12.24 seconds per report in the 0-shot setting, while DeepSeek-R1 needed 92.16 seconds. The fastest and slowest radiologists required 109.44 seconds and 192.96 seconds per report, respectively. In the few-shot setting, LLMs showed a slight decrease in processing time and remained substantially faster than human experts (Figure S3 in Multimedia Appendix 1).

## Discussion

### Principal Findings

This study evaluated the performance of DeepSeek-R1 in detecting errors in Chinese emergency radiology reports. We compared its accuracy and processing efficiency with Grok3 and with radiologists of different experience levels under both 0-shot and few-shot settings. By introducing a real-world, emergency-focused evaluation framework, this work provides methodological insights into AI-assisted quality control in digital health care.

DeepSeek-R1 demonstrated strong error detection performance in emergency radiology. Under stress-testing conditions, it achieved a detection rate of 84.4% in the few-shot setting, indicating high sensitivity to predefined error types rather than direct real-world detection rates. Notably, its performance exceeded that of resident radiologists in identifying critical errors, supporting its potential clinical value for improving report accuracy and facilitating timely patient management.

### Comparison With Previous Studies

Previous studies have shown that LLMs, such as GPT-4, can detect errors in radiology reports. Most of these studies relied on synthetically generated error datasets across imaging modalities, including radiography, CT, and MRI. Our study extends this literature in several important ways.

First, to our knowledge, this is the first validation of an LLM specifically in the emergency radiology setting. Emergency radiology is a high-risk environment characterized by time pressure, incomplete information, and urgent clinical decision-making, which differs substantially from routine radiologic workflows.

Second, we tailored the model to Chinese clinical language, particularly to address spelling and semantic ambiguities introduced by Pinyin-based input. This addresses a key limitation in prior cross-lingual applications and is especially relevant given reports of the limited accuracy of models such as Claude 3.5 in Chinese ultrasound reports. Third, by analyzing 7435 real-world clinical reports across CT, MRI, and radiography, our study avoided exclusive reliance on synthetic error injection [12,27]. This approach better captures the complexity and variability of real emergency reporting workflows.

Together, these findings suggest that DeepSeek-R1 may serve as an efficient and scalable assistant for radiologists, particularly for identifying critical omissions and communication-related errors. Importantly, validation using independent datasets from 2 additional tertiary hospitals with different reporting systems supports the robustness of the proposed approach across institutions and report templates.

Consistent with prior studies, our results indicate that LLMs can proofread radiology reports at a level comparable to most human readers. Reporting errors occur across all levels of radiologist experience [21,23], and the observed performance likely reflects routine clinical practice. These findings highlight the potential role of AI in supporting radiology workflows beyond image interpretation alone [28,29].

In terms of efficiency, our results align with previous studies examining the integration of LLMs into radiologic workflows [30,31]. When used as a proofreading tool, DeepSeek-R1 achieved performance comparable to human readers while requiring less time. Unlike human readers, whose performance may be affected by multitasking or fatigue during off-hours [32-34], DeepSeek-R1 provides stable and consistent output independent of such factors.

### Limitations

This study has several limitations. First, part of the evaluation used predefined correct and incorrect report sets in which 127 errors were deliberately introduced. Although the error categories were derived from commonly reported patterns, this synthetic design cannot fully capture the diversity and contextual complexity of errors in routine clinical practice. Our real-world validation in stage 4 provides a reference for naturalistic error frequency. Among 800 consecutive emergency radiology reports, 117 true reporting errors were identified, corresponding to an overall error rate of 14.6%, with omission being the most common error type. This prevalence is lower than the error density used in the synthetic datasets in stages 2‐3. The higher synthetic error density was intentionally adopted to create a controlled and sufficiently challenging evaluation environment. This design allowed statistically efficient comparison of error detection performance across AI models and human readers within a limited sample size. Accordingly, performance metrics from stages 2‐3 should be interpreted as reflecting stress-tested detection capability rather than real-world error prevalence. Although real-world validation yielded a moderate PPV of 56.5%, this result should be interpreted in the context of workflow efficiency. In our study, AI-assisted report review was faster than human-only proofreading. When false-positive alerts can be dismissed with brief verification, the additional workload introduced by false positives remains limited, and overall time efficiency may be preserved. Moreover, PPV is inherently dependent on error prevalence and should not be extrapolated across settings with different baseline error rates. From a workflow perspective, AI assistance shifts the task from exhaustive manual proofreading to alert-based verification. Despite the presence of false positives, this approach reallocates radiologists’ cognitive effort from error searching to decision confirmation. This shift may be particularly valuable in high-fatigue emergency settings.

Second, the exploratory analyses in stages 2‐3 were conducted in a simulated environment that differed from routine clinical practice. Factors such as artificially high error prevalence, lack of imaging data, long report length, and task instructions optimized for AI may have influenced reader performance. In addition, learning effects from repeated exposure to similar error patterns may have biased results. These factors should be considered when interpreting human-AI comparisons.

Third, the error taxonomy and definitions of false-positive subtypes involve some subjectivity. Although granular categorizations were applied to reduce bias, certain subtypes may not fully reflect errors encountered in routine practice. Subgroup analyses of rare error categories were also limited by small sample sizes and should be considered exploratory.

Fourth, although this study assessed time efficiency, it did not include a formal cost-effectiveness analysis. Reduced processing time suggests potential resource savings, but costs related to infrastructure, system integration, and maintenance were not evaluated.

Fifth, the experimental setting may have introduced a Hawthorne effect [35], whereby radiologists’ awareness of observation temporarily enhanced performance. This may have led to overestimation of human error detection rates. Importantly, this does not undermine the clinical relevance of our findings. DeepSeek-R1 consistently outperformed resident radiologists, even under conditions likely to favor human readers, suggesting that its error detection capability is robust and may be more pronounced in routine, nonobserved clinical settings.

Finally, generalizability warrants consideration. Although this study included data from 3 tertiary hospitals with different reporting systems and template styles, all institutions were within the same health care system. Validation using dataset 2 supports robustness across institutions within Chinese emergency radiology; however, further validation in other hospitals and nonemergency settings is needed, particularly in environments with lower baseline error prevalence. In addition, the strong performance of DeepSeek-R1 is closely related to its optimization for Chinese clinical language. Direct 0-shot transfer to other languages may therefore be inappropriate, and language-specific optimization or models with stronger multilingual medical pretraining will likely be required.

Overall, while our findings support the robustness of DeepSeek-R1 across institutions and report templates within Chinese emergency radiology, generalization to other clinical settings or languages should be approached cautiously. Further prospective validation and language-specific optimization are required. Additional domain-specific fine-tuning using emergency medicine–focused corpora may further improve performance in specialized settings such as trauma or neurologic imaging.

### Conclusion

DeepSeek-R1 represents a meaningful advance in automated quality control for radiology reports, particularly in Chinese emergency settings. Its ability to identify clinically significant errors with high efficiency supports its role as an assistive proofreading tool in modern radiology workflows.

## Supplementary material

10.2196/86841 Multimedia Appendix 1 Example for metric calculation. Table S1. Detailed prompts parameters of large language models used in this study; Table S2. Clinical impact and definition of false positive responses generated by DeepSeek-R1; Table S3. Example dataset for metric calculation; Table S4. Interrater agreement between LLMs and radiologists in a 0-shot setting; Table S5. Interrater agreement between LLMs and radiologists in a few-shot setting; Figure S1. The examples of various errors; Figure S2. Distribution of the 5 error types for both real and artificial errors (datasets 1 and 2); and Figure S3. Time efficiency analysis. Bar graphs comparing reading time for error detection for the large language models and radiologists.

## Abbreviations

AI: artificial intelligence
CT: computed tomography
FPRR: false positive report rate
LLM: large language model
MRI: magnetic resonance imaging
PPV: positive predictive value
TPR: true positive rate
