# Supplementary material for: Error Detection in Emergency Radiology Reports Using a Large Language Model: Multistage Evaluation Study
Source: J Med Internet Res. 2026 Apr 14;28:e86841. doi: 10.2196/86841 (PMC13078523; doi:10.2196/86841)

**Example for Metric Calculation.**

Calculation Steps:

1. Build confusion matrix

​The confusion matrix is a table used to evaluate the performance of a classification model. It summarizes the model's predictions by comparing them to the actual labels, categorizing the outcomes into four key types: True Positives (TP), False Positives (FP), False Negatives (FN), and True Negatives (TN). These four elements form the mathematical foundation for deriving essential performance metrics such as Positive Predictive Value (PPV), True Positive Rate (TPR), F1 score, False Positive Report Rate (FPRR).

2. Measure related indicators

PPV, TPR, F1 score, FPRR are the essential measures to measure the capability of model in Errors Detecting. The define is as below

PPV: PPV measures the proportion of actual positives among all positive results detected by the model. A high PPV indicates that most of the errors detected by the model are true errors.

𝑃𝑃𝑉 = 𝑇𝑃/𝑇𝑃 + 𝐹𝑃

TPR: Also known as Recall, TPR measures the proportion of actual error reports correctly identified by the model. A high TPR indicates that the model can detect most of the actual errors.

𝑇𝑃𝑅 =𝑇𝑃/𝑇𝑃 + 𝐹𝑁

F1 Score: The F1 Score is the harmonic mean of PPV and TPR, combining both precision and sensitivity. A high F1 Score indicates that the model performs well in terms of both accuracy and sensitivity.

F1 𝑆𝑐𝑜𝑟𝑒 = 2 × 𝑃𝑃𝑉 × 𝑇𝑃𝑅/ (𝑃𝑃𝑉 + 𝑇𝑃𝑅)

FPRR: This metric reflects the proportion of reports incorrectly detected as having errors among all reports.

𝐹𝑃𝑅𝑅 = 𝐹𝑃 reports/Total Number of Reports

In this example, the statistic of each report was provided by Table S1. According to the table, the confusion matrix was described as:
 TP: 1+2+1+1=5

FP: 1+1+1=3

FN: 1+1+1+2=5

TN: 1

In this test, confusion matrix in last step was used to calculate these metrics

PPV: 5 / 5+3 = 0.625

TPR: 5 / 5+5 ≈ 0.500

F1 Score: 2×0.625×0.500 / (0.625+0.500) ≈ 0.556

FPRR: 3 / 8 = 0.378

**Table S1.** The anonymized example reports used for few-shot prompting.

| **One error-free report and six reports containing the various categories of errors listed** | **Radiological Report** | **Error and Error Type(s)** |
| --- | --- | --- |
| 1 | **Findings:**  In the distal phalanx of the left ring finger, there are multiple radiolucent fracture lines consistent with comminution. The distal fracture segment demonstrates dorsal displacement. Associated soft tissue swelling is noted, with several small osseous fragments in the surrounding soft tissues. The remaining phalanges are intact, with normal osseous mineralization. Interphalangeal joints are well aligned, and joint spaces are preserved.  **Impression:**  Comminuted fracture of the distal phalanx of the left ring finger with dorsal displacement of the distal fragment. | **Error:** No error  **Error type(s):** No error |
| 2 | **Findings:**  Normal alignment and vertebral body height are preserved throughout the lumbar spine, except at L5. The L5 vertebral body demonstrates mild anterior translation relative to S1, consistent with grade I anterolisthesis. Bilateral pars interarticularis defects are present at L5, in keeping with spondylolysis. The intervertebral discs from L1 through S1 show normal height and signal intensity, without evidence of significant bulging or herniation. The thecal sac and exiting nerve roots are intact, with no evidence of compression. Epidural fat planes are maintained. The bony spinal canal demonstrates normal calibre without stenosis.  **Impression:**  Grade I anterolisthesis of L5.  No disc herniation, nerve root compression, or spinal canal stenosis. | **Error:** The ‘findings’ describe grade I anterolisthesis of L5 accompanied by bilateral pars interarticularis defects. The ‘impression’, however, reports only the anterolisthesis and omits mention of the pars defects.  **Error type(s):** Omission (with vs without) |
| 3 | **Findings:**  Adjacent to the middle phalanx of the left fifth finger, there is a small, well-defined osseous density fragment. Surrounding soft tissue swelling is noted. The remaining osseous structures of the left hand are intact, without evidence of displaced fracture. The joints are normally aligned, the cortical articular surfaces are smooth, and the joint spaces are preserved.  **Impression:**  Small osseous density adjacent to the right fifth middle phalanx, suspicious for an avulsion fracture, with associated soft tissue swelling. Recommend correlation with clinical examination and, if indicated, further evaluation with CT. | **Error: I**n the ‘Findings’ section, the location was recorded as “left fifth middle phalanx,” while in the ‘Impression’ it was documented as “right fifth middle phalanx.”  **Error type(s):** Side confusion (left vs right). |
| 4 | **Findings:**  Disruption of cortical continuity is noted in the distal phalanx of the left hallux (拇趾), with an identifiable transverse/radiolucent fracture line. There is mild displacement of the fracture fragments, but overall alignment remains acceptable. Surrounding soft tissue swelling is observed.  The remaining metatarsals and phalanges show normal alignment and bony integrity, with smooth articular surfaces and preserved joint spaces. No additional displaced fracture is identified.  **Impression:**  Mildly displaced fracture of the distal phalanx of the left hallux (拇指), with associated soft tissue swelling. | **Error:** The Chinese term “拇趾” in the Findings was mistakenly typed as “拇指”.  **Error type(s):** Spelling error |
| 5 | **Findings:**  There is a fracture at the inferior pole of the patella with displacement of the larger fragment inferiorly and anteriorly, and displacement of a smaller bony fragment inferiorly. Alignment within the right knee joint is appropriate. No further pathologic step-offs or interruptions of the cortex are delineated. The bone trabeculae are homogeneously structured. The visualized soft tissues are unremarkable.  **Impression:**  Nondisplaced fracture at the superior pole of the patella. | **Error:** The 'Findings' describe a displaced fracture at the inferior pole of the patella. However, the 'Impression' states a nondisplaced fracture at the superior pole of the patella.  **Error type(s):**  Insertion (displaced vs nondisplaced). Side confusion (inferior vs superior). |
| 6 | **Findings:**  The liver demonstrates smooth contours and normal proportions. Multiple rounds, low-attenuation lesions are present within the hepatic parenchyma, with well-defined margins. The largest lesion measures approximately 1.2 cm in diameter. No intrahepatic or extrahepatic bile duct dilatation is noted. The gallbladder is normal in shape, with no abnormal intraluminal density.  The pancreas, spleen, and both kidneys appear normal in morphology, and unenhanced images reveal no abnormal attenuation lesions. The stomach is adequately distended, with no apparent mural thickening. The walls of the colon and rectum show no significant thickening; no definite mass is seen within the small bowel. The appendix is normal in appearance, without abnormal intraluminal density.  The uterus and bilateral adnexal regions appear normal on unenhanced imaging, without abnormal attenuation lesions. The urinary bladder is adequately distended, with no mural thickening and no abnormal intraluminal density.  No significantly enlarged lymph nodes are identified in the retroperitoneum or along the para-aortic region.  **Impression:**  Multiple low-attenuation hepatic lesions, largest measuring approximately 1.2 mm.  Recommend contrast-enhanced CT for further evaluation if clinically indicated. | **Error:** In the 'Findings', the largest hepatic lesion was documented as measuring approximately 1.2 cm in diameter. However, the 'Impression' states it as 1.2 mm in diameter.  **Error type(s):** Other error (centimeter vs millimeter) |

**Table S2.** Clinical impact and definition of false positive responses generated by FeepSeek-R1.

| **Criteria** | **Definition** |
| --- | --- |
| Erroneous False  Positive | Implementing the model’s revision advice results in the introduction of errors  into the report. |
| Serious False  Positive | Implementing the model’s revision advice does not result in errors but has the  potential to diminish the report’s comprehensibility or clarity, consequently  degrading its quality. |
| Neutral False  Positive | Implementing the model’s revision advice neither introduces errors nor  changes the overall quality of the report. |
| Potentially Beneficial  False Positive | Implementing the model’s revision advice does not result in errors and has the  potential to improve the report’s comprehensibility or clarity, potentially  enhancing its quality. |

| **Table S3.** Example dataset for metric calculation. | | | | | | |
| --- | --- | --- | --- | --- | --- | --- |
| **Report Number** | **Actual Error Count** | **Count of Errors**  **Detected by Model** | **True Positives** | **False Positives** | **False Negatives** | **True Negatives** |
| 1 | 0 | 0 | 0 | 0 | 0 | 1 |
| 2 | 1 | 0 | 0 | 0 | 1 | - |
| 3 | 1 | 1 | 1 | 0 | 0 | - |
| 4 | 1 | 1 | 0 | 1 | 1 | - |
| 5 | 2 | 2 | 2 | 0 | 0 | - |
| 6 | 2 | 2 | 1 | 1 | 1 | - |
| 7 | 2 | 0 | 0 | 0 | 2 | - |
| 8 | 1 | 2 | 1 | 1 | 0 | - |

| **Table S4.** Interrater agreement between LLMs and radiologists in zero-shot setting. | | | | | | | | |
| --- | --- | --- | --- | --- | --- | --- | --- | --- |
| Reader | Deepseek-R1 | Grok3 | Senior 1 | Senior 2 | Attending 1 | Attending 2 | Resident 1 | Resident 2 |
| Deepseek-R1 | ... | 0.45 | 0.45 | 0.57 | 0.49 | 0.46 | 0.42 | 0.49 |
| Grok3 | 0.45 | ... | 0.49 | 0.37 | 0.15 | 0.23 | 0.28 | 0.23 |
| Senior 1 | 0.45 | 0.49 | ... | 0.32 | 0.29 | 0.21 | 0.21 | 0.36 |
| Senior 2 | 0.57 | 0.37 | 0.32 | ... | 0.49 | 0.31 | 0.31 | 0.46 |
| Attending 1 | 0.49 | 0.15 | 0.29 | 0.49 | ... | 0.30 | 0.30 | 0.33 |
| Attending 2 | 0.46 | 0.23 | 0.21 | 0.31 | 0.30 | ... | 0.66 | 0.26 |
| Resident 1 | 0.42 | 0.28 | 0.21 | 0.31 | 0.30 | 0.66 | ... | 0.13 |
| Resident 2 | 0.49 | 0.23 | 0.36 | 0.46 | 0.33 | 0.26 | 0.13 | ... |
| Note.—Data are Cohen κ values (0.01–0.20, none to slight agreement; 0.21–0.40, fair agreement; 0.41–0.60, moderate agreement; 0.61–0.80, substantial agreement; and 0.81–1.00, almost perfect agreement. LLMs: large language models. | | | | | | | | |

| **Table S5.** Interrater agreement between LLMs and radiologists in few-shot setting. | | | | | | | | |
| --- | --- | --- | --- | --- | --- | --- | --- | --- |
| Reader | Deepseek-R1 | Grok3 | Senior 3 | Senior 4 | Attending 3 | Attending 4 | Resident 3 | Resident 4 |
| Deepseek-R1 | ... | 0.29 | 0.09 | 0.45 | 0.35 | 0.08 | 0.27 | 0.11 |
| Grok3 | 0.29 | ... | 0.45 | 0.44 | 0.61 | 0.05 | 0.52 | 0.37 |
| Senior 3 | 0.09 | 0.45 | ... | 0.22 | 0.45 | 0.21 | 0.37 | 0.07 |
| Senior 4 | 0.45 | 0.44 | 0.22 | ... | 0.44 | 0.04 | 0.30 | 0.30 |
| Attending 3 | 0.35 | 0.61 | 0.45 | 0.44 | ... | 0.02 | 0.61 | 0.33 |
| Attending 4 | 0.07 | 0.05 | 0.21 | 0.04 | 0.02 | ... | 0.03 | 0.04 |
| Resident 3 | 0.27 | 0.52 | 0.37 | 0.30 | 0.61 | 0.03 | ... | 0.15 |
| Resident 4 | 0.11 | 0.37 | 0.07 | 0.30 | 0.33 | 0.04 | 0.15 | ... |
| Note.—Data are Cohen κ values (0.01–0.20, none to slight agreement; 0.21–0.40, fair agreement; 0.41–0.60, moderate agreement; 0.61–0.80, substantial agreement; and 0.81–1.00, almost perfect agreement. LLMs: large language models. | | | | | | | | |

**Figure S1.** The examples of various errors.

Original Radiology Report and Examples of Five Error Categories, (A) Chinese radiology report template, (B) English radiology report template, (C) Examples of the five types of errors in reports, the left image shows Insertion error (highlighted with red) and Side confusion error (highlighted with blue), while the right image shows ltem omission error (highlighted with red), Spelling error (highlighted with blue) and Other errors (highlighted with yellow).


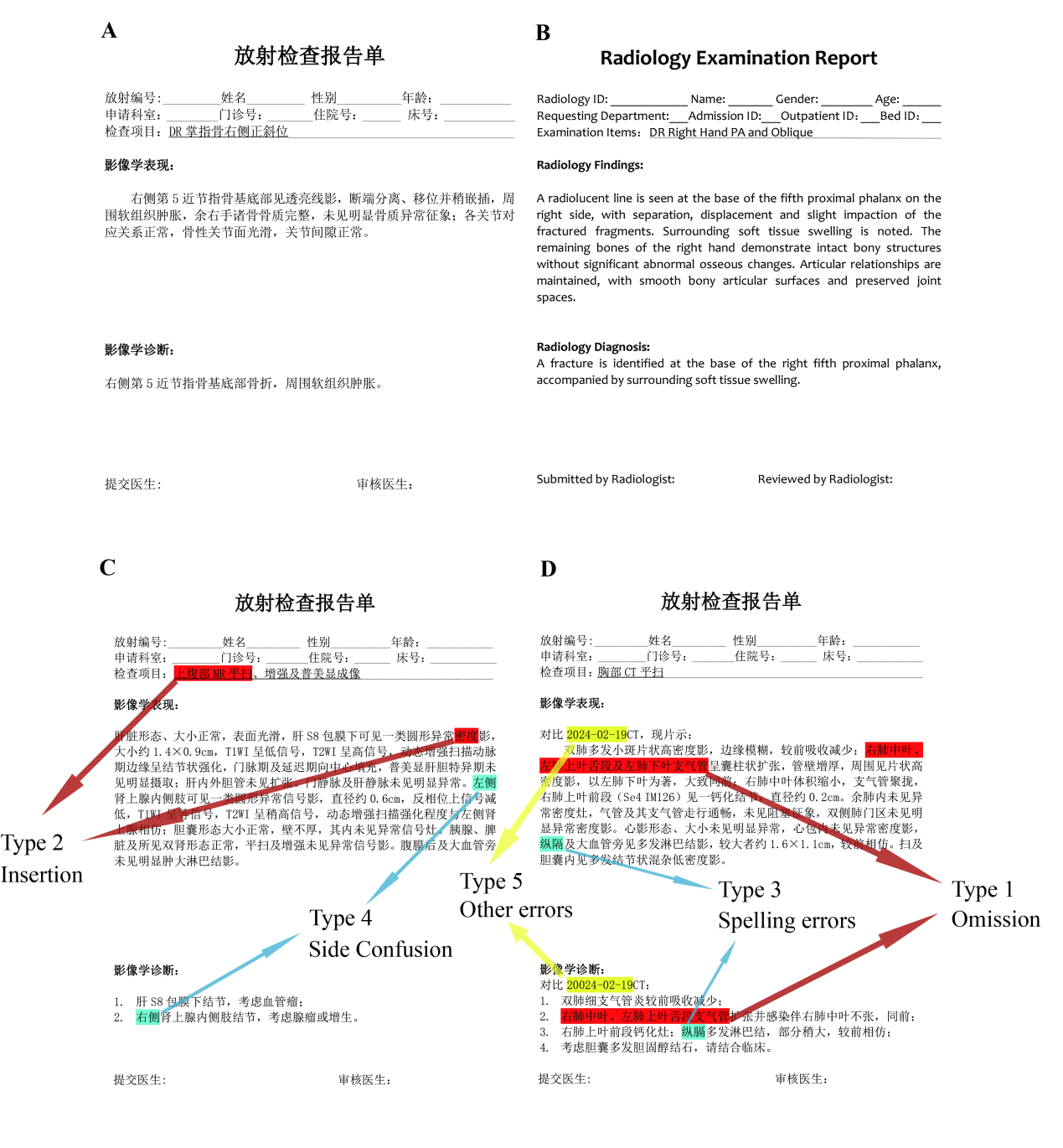


**Figure S2.** Distribution of the five error types for both real and artificial errors (datasets 1 and 2). The bar chart demonstrates the distribution of error types in both conditions. It can be observed that the relatively common types of errors (1, 2, and 3) account for a relatively high proportion of errors in both conditions, especially for Item omission. Furthermore, the errors observed in the real condition were more complex, resulting in the presence of a greater number of the six identified error types. The five error types are as follows: 1 = Item omission; 2 = Insertion; 3= Spelling errors; 4 = Side Confusion; 5 = Other errors.


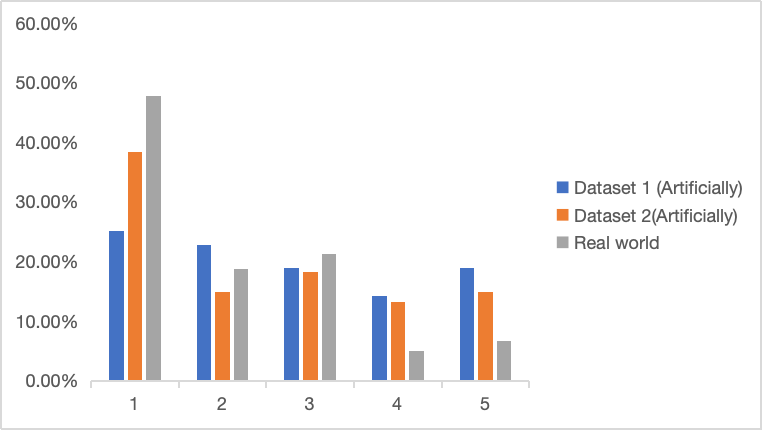


**Figure S3.** Time efficiency analysis. Bar graphs comparing reading time for error detection for the large language models and radiologists. **(A)** Total reading time (hours) in zero-shot setting. **(B)** Total reading time (hours) in few-shot setting.


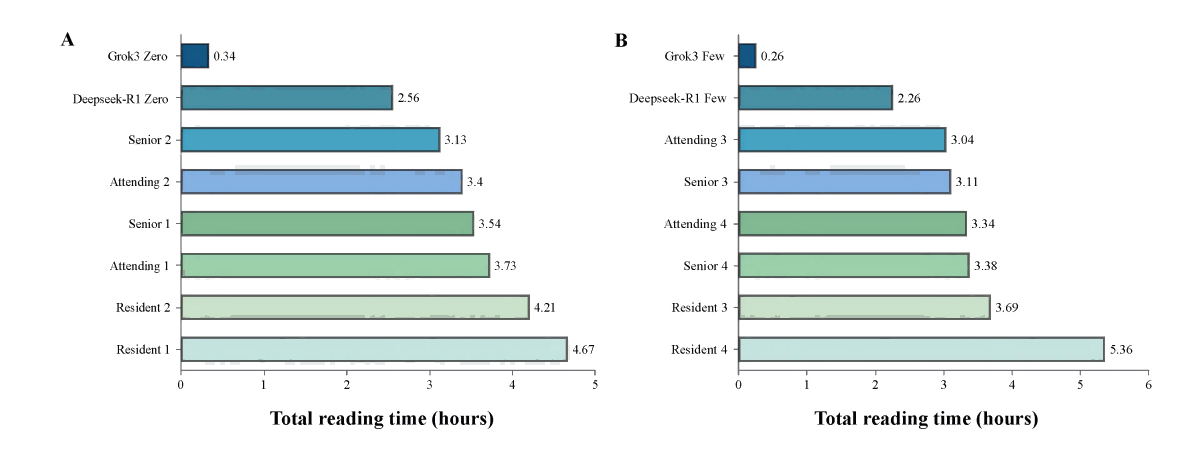

Supplement: Multimedia Appendix 1 — Example for metric calculation. Table S1. Detailed prompts parameters of large language models used in this study; Table S2. Clinical impact and definition of false positive responses generated by DeepSeek-R1; Table S3. Example dataset for metric calculation; Table S4. Interrater agreement between LLMs and radiologists in a 0-shot setting; Table S5. Interrater agreement between LLMs and radiologists in a few-shot setting; Figure S1. The examples of various errors; Figure S2. Distribution of the 5 error types for both real and artificial errors (datasets 1 and 2); and Figure S3. Time efficiency analysis. Bar graphs comparing reading time for error detection for the large language models and radiologists. [file jmir-v28-e86841-s001.docx]
